# Bleeding from ruptured hepatic metastases as a cause of syncope in an octogenarian: a case report

**DOI:** 10.1186/1752-1947-4-194

**Published:** 2010-06-26

**Authors:** Ian W Seetho, Simon Stinchcombe, Mazen M Rizeq

**Affiliations:** 1 Department of Medicine, City Hospital, Nottingham, Nottingham University Hospitals NHS Trust. Hucknall Road. Nottingham NG5 1PB, UK; 2 Department of Radiology, King's Mill Hospital, Sherwood Forest Hospitals NHS Foundation Trust, Mansfield Road. Nottingham NG17 4JL, UK; 3 Department of Stroke Medicine and Medicine for the Elderly, King's Mill Hospital, Sherwood Forest Hospitals NHS Foundation Trust, Mansfield Road. Nottingham NG17 4JL, UK

## Abstract

**Introduction:**

Acute hemoperitoneum as a result of hemorrhage from liver metastases is an uncommon but serious condition. The use of appropriate imaging is important in the diagnosis and can have a profound impact on subsequent management. This case is important because the presentation was of recurrent syncopal episodes with an unusual underlying cause. This case highlights the need to consider this diagnosis in the differential in patients presenting with collapse in the acute setting.

**Case presentation:**

We present the case of an 85-year-old Caucasian man who was admitted following a collapse episode and was found to be persistently hypotensive despite aggressive resuscitation. An acute intra-peritoneal bleed originating from hepatic metastases from an unknown primary was identified promptly with computed tomography imaging and was subsequently managed conservatively.

**Conclusions:**

This case aims to convey key teaching points: (A) the need to consider intra-abdominal hemorrhage in the differential diagnosis when assessing patients with collapse; and (B) the use of appropriate imaging such as computed tomography can facilitate a prompt diagnosis and appropriate management steps can then be taken accordingly.

## Introduction

Spontaneous rupture of hepatic metastases leading to hemoperitoneum may initially present as collapse in the elderly and is a serious diagnosis. In this case report, we present a patient who was admitted following recurrent syncopal episodes with clinical features of persistent hypotension. A sudden fall in his hemoglobin level suggested that an acute bleed had led to his collapse. This was an important investigation finding in determining the cause of his syncopal episodes. The underlying diagnosis of acute hemorrhage from liver metastases was confirmed on computed tomography (CT) imaging.

## Case presentation

An 85-year-old Caucasian man was admitted to hospital following three collapse episodes with transient loss of consciousness at home. Each episode was short-lived lasting several minutes. Apart from mild abdominal generalized discomfort, there were no other symptoms. There was no history of recent trauma. He had no history of similar episodes but was known to have severe aortic stenosis, type 2 diabetes, paroxysmal atrial fibrillation, hypertension and a previous duodenal ulcer bleed.

At that time, he was taking aspirin, bisoprolol, omeprazole and ramipril. He lived with his daughter and was independent with his activities of daily living. He had not smoked for 35 years and his alcohol consumption was minimal.

On examination, he was apyrexial, oxygen saturation was 100% on air. His blood pressure was 80/40 mmHg. He was persistently hypotensive despite aggressive fluid resuscitation. There was an ejection systolic murmur on cardiac auscultation. His venous pressure was not elevated and there was no leg edema. The lungs were clear on auscultation. Upper and lower limb pulses were equal bilaterally. Examination of his abdomen revealed mild epigastric discomfort, but there was no rebound or peritonism and bowel sounds were present. Per rectal examination was normal.

Initial blood results showed a hemoglobin of 11.3 (13-18 g/dL), white cell count of 11 (4-11 × 10^9^/L), and platelets of 136 (150-450 × 10^9^/L). Coagulation profile, renal function and liver function tests were within normal limits. His chest radiograph was normal and his electrocardiogram showed left ventricular hypertrophy. At admission, he was taken to the coronary care unit for cardiac monitoring because of the history of collapse with loss of consciousness which was thought to be related to his aortic stenosis. An urgent echocardiogram was performed which showed evidence of aortic stenosis, but no evidence of critical stenosis with good ejection fraction > 55% and good biventricular contraction.

A repeat full blood count showed that his hemoglobin had fallen to 4.9 g/dL and he was transfused with red cells, platelets and cryoprecipitate. The impression was that this patient had a possible dissecting thoracic aneurysm that was possibly extending into the abdomen. He was transferred to the intensive care unit.

**Figure 1:**
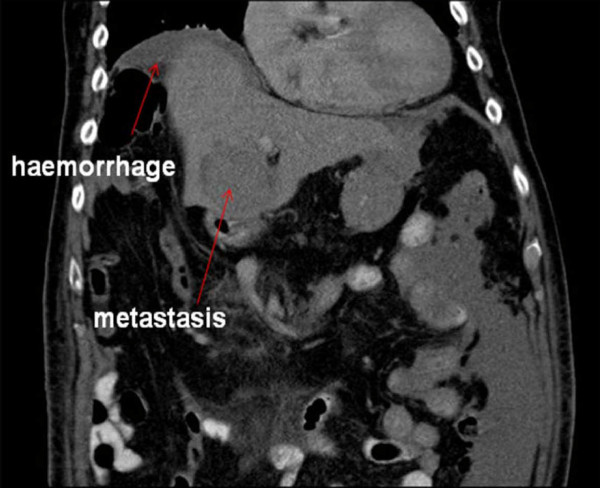
**Coronal computed tomography view of the patient showing intra-abdominal hemorrhage and liver metastasis (red arrows)**.

**Figure 2:**
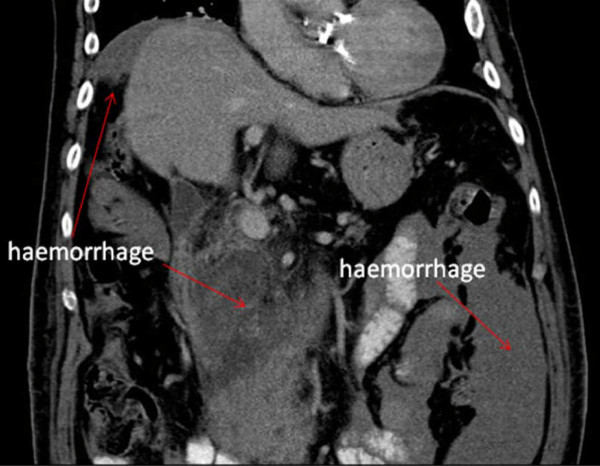
**Coronal computed tomography view of the patient showing intra-abdominal hemorrhage (red arrows)**.

**Figure 3:**
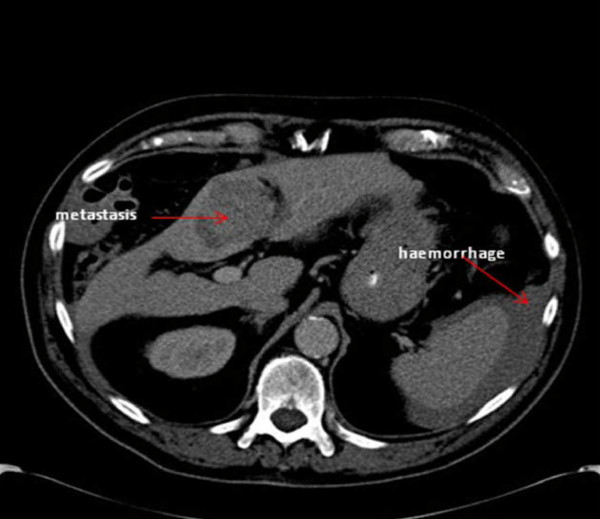
**Axial computed tomography view of the patient showing metastasis and intra-abdominal hemorrhage (red arrows)**.

In view of the differential diagnosis of a possible dissection, an urgent chest and abdomen CT scan was performed which showed normal appearances of the thoracic and abdominal aorta with no evidence of aneurysm or dissection. However, the scan revealed a large amount of free intra-peritoneal fluid with areas of low attenuation in the right lobe of the liver. The appearances were concluded to be of metastatic disease within the liver (Figures 1, 2, 3. No primary tumor was identified. A diagnostic peritoneal tap was performed and frank blood was aspirated confirming that there was hemoperitoneum. An acute intra-abdominal bleed from the liver metastatic disease was diagnosed.

Our patient had an esophageal gastro-duodenal endoscopy as he had been taking aspirin and had a past history of a duodenal ulcer. This did not show any evidence of bleeding. A rigid sigmoidoscopy was also normal.

Whilst on the intensive care ward, our patient's blood pressure subsequently improved and he did not require inotropic support. He had no further hypotensive episodes and improved during his stay on the ward. Given the advanced nature of his hepatic metastases, he did not wish to have further investigations to identify the primary source of the metastases and decided on conservative supportive treatment, as advised by the oncologists. He was referred to the Macmillan and Hospital Palliative Care Team. Subsequently, he died three months later. A post-mortem was not performed.

## Discussion

Tumor perforation and bleeding may occur as a complication of primary hepatocellular carcinoma [1]. This complication is not uncommon in primary hepatocellular carcinoma [2]. The significance of this case is that we describe acute rupture of hepatic metastases resulting in acute hemoperitoneum that initially presented as a syncopal episode. There are only a few reported cases in the literature of acute hemoperitoneum secondary to the rupture of liver metastases [3-9] originating from different sources. These sources include nasopharyngeal cancer [5], gastric cancer [6], lung cancer [7], renal cell carcinoma [8] and carcinoma of the liver [9].

Intra-peritoneal hemorrhage frequently presents with acute abdominal pain and can be life-threatening. CT is commonly used as an imaging modality in the investigations of these patients, but ultrasound and magnetic resonance imaging may also be used in the diagnosis [10]. It should be noted that in this case, our patient presented following recurrent collapse episodes rather than with an acute abdomen. He did not wish for further investigations to identify the primary source given the advanced nature of the liver metastases. As such, the source of his liver metastases was not identified. He survived the acute bleeding episode with conservative management alone.

The literature reports laparotomy as a management option for metastatic hepatic hemorrhage but this would entail surgical risks for the patient [11,12]. In hemoperitoneum occurring as a result of rupture of hepatocellular carcinoma, transcatheter arterial embolization has been previously described as a potential therapeutic option [1,2].

## Conclusions

In conclusion, a high index of suspicion is needed in the acute setting when considering the possibility of spontaneous hemoperitoneum in a patient who presents with syncope, especially with an acute abdomen. This is particularly important if there is a known history of neoplastic process. This case highlights an unusual source of intra-abdominal bleeding which was from liver metastatic disease. The use of appropriate CT imaging in this case facilitated the prompt diagnosis and subsequent management steps were then taken accordingly.

## Consent

Written informed consent was obtained from our patient's next-of-kin for publication of this case report and any accompanying images. A copy of the written consent is available for review by the Editor-in-Chief of this journal.

## Competing interests

The authors declare that they have no competing interests.

## Authors' contributions

All authors contributed equally to the manuscript.
